# Metformin ameliorates experimental-obesity-associated autoimmune arthritis by inducing FGF21 expression and brown adipocyte differentiation

**DOI:** 10.1038/emm.2017.245

**Published:** 2018-01-26

**Authors:** Eun-Kyung Kim, Seung Hoon Lee, Seon-Young Lee, Jae-kyung Kim, Joo Yeon Jhun, Hyun Sik Na, Se-Young Kim, Jong Young Choi, Chul Woo Yang, Sung-Hwan Park, Mi-La Cho

**Affiliations:** 1 The Rheumatism Research Center, Catholic Research Institute of Medical Science, College of Medicine, The Catholic University of Korea, Seoul, South Korea; 2 Division of Hepatology, Department of Internal Medicine, College of Medicine, Seoul St. Mary’s Hospital, The Catholic University of Korea, Seoul, Republic of Korea; 3 Division of Nephrology, Department of Internal Medicine, Seoul St. Mary's Hospital, College of Medicine, The Catholic University of Korea, Seoul, Republic of Korea; 4 Divison of Rheumatology, Department of Internal Medicine, College of Medicine, Seoul St. Mary’s Hospital, The Catholic University of Korea, Seoul, Republic of Korea

## Abstract

Rheumatoid arthritis (RA) is a systemic autoimmune disease involving excessive inflammation. Recently, RA associated with a metabolic disorder was revealed to be non-responsive to RA medications. Metformin has been reported to have a therapeutic effect on RA and obesity. The aim of this investigation was to study the therapeutic effect and the underlying mechanism of metformin's action in an experimental model of collagen-induced arthritis (CIA) associated with obesity. Metformin was administered daily for 13 weeks to mice with CIA that had been fed a high-fat diet. Metformin ameliorated the development of CIA in obese mice by reducing autoantibody expression and joint inflammation. Furthermore, metformin decreased the expression levels of pSTAT3 and pmTOR and had a small normalizing effect on the metabolic profile of obese CIA mice. In addition, metformin increased the production of pAMPK and FGF21. Metformin also induced the differentiation of brown adipose tissue (BAT), which led to a reciprocal balance between T helper (Th) 17 and regulatory T (Treg) cells *in vitro* and *in vivo*. These results suggest that metformin can dampen the development of CIA in obese mice and reduce metabolic dysfunction by inducing BAT differentiation. Thus, metformin could be a therapeutic candidate for non-responsive RA.

## Introduction

Rheumatoid arthritis (RA), a progressive and systemic form of autoimmune arthritis, is characterized by chronic inflammation and the infiltration of synovial immune cells into the affected joints. It has been well-documented that several proinflammatory cytokines are involved in the pathogenesis of RA and exacerbate its progression.^1^ Although there have been advances in the therapeutic functions of RA drugs, the treatment of RA remains extremely difficult.^2, 3^ Furthermore, a significant proportion of RA patients (30–40%) have no response to RA drugs such as anti-tumor necrosis factor (TNF)-α antibody therapy (such as infliximab).^4, 5^ Currently, RA patients who show no response to RA drugs are more susceptible to non-response to other biologics used to treat RA.^6^ It has recently been reported that the probability of non-response to RA treatment is associated with metabolic disorders.^7^ Obesity, a metabolic disorder that plays a key role in the inflammatory response, leads to the upregulation of proinflammatory cytokine expression. It has been demonstrated that there is in increase in the expression of proinflammatory cytokines and a parallel decrease in the expression of anti-inflammatory cytokines among obese individuals compared to healthy lean individuals.^8, 9^ Additionally, obesity is associated with inflammatory and autoimmune diseases, thus increasing the possibility of diseases such as RA occurring in obese people.^10, 11^

Brown adipose tissue (BAT) is found predominantly in human neonates and has an important role in the modulation of body temperature. It has been demonstrated that BAT is an essential factor in non-shivering thermogenesis and energy production, and that it can therefore slow the progression of diet-induced obesity.^12^ The development of metabolic disorders has been shown to be inhibited by BAT and several genes related to BAT differentiation, making BAT a promising candidate for the treatment of metabolic disorders.^13^

Fibroblast growth factor (FGF) 21 is a metabolic hormone that is primarily expressed by the liver, but is also released by adipocytes. It has been demonstrated that FGF21 has a significant role in the progression of obesity.^14^ Indeed, obesity is characterized by resistance to FGF21^15^ and there is notable interest in the administration of FGF21 as a therapy for obesity.^16^ Recently, FGF 21 production was shown to be associated with BAT differentiation. The circulating level of FGF21 and the activity of BAT were increased during acute cold exposure in human subjects.^17^ FGF21 also demonstrated potential therapeutic function in mice with collagen-induced arthritis (CIA).^18^

Metformin is a biguanide anti-diabetic drug. In patients with type 2 diabetes, body weight decreases with metformin treatment.^19, 20^ Recently, metformin has demonstrated therapeutic efficacy in other disorders. It has been suggested that metformin has the ability to ameliorate experimental autoimmune arthritis and colitis by decreasing the activation of signal transducer and activator of transcription (STAT) 3.^21, 22^ Metformin has also been shown to have a therapeutic effect in obese mouse models via the upregulation of genes involved in BAT differentiation.^23^ Moreover, the expression of FGF21 was shown to increase in obese mice following treatment with metformin.^23^

We hypothesized that metformin would attenuate the development of CIA in mice with high-fat diet-induced obesity. The present study sought to determine whether metformin ameliorates CIA in obese mice by upregulating FGF21 expression and BAT differentiation. We measured the therapeutic and anti-inflammatory activity of metformin in obese CIA mice by studying its activity on the Th17/Treg balance and on BAT differentiation *in vitro* and *in vivo*.

## Materials and methods

### Animals

Five 7-week-old male DBA/1J mice were purchased from Orient Bio. The mice were housed in polycarbonate cages and fed 60 kcal fat-derived calories or standard mouse chow (Ralston Purina, St Louis, MO, USA) and water *ad libitum*. All experimental procedures were examined and approved by the Animal Research Ethics Committee of the Catholic University of Korea (permit number: CUMC:2015-0009-01), which conforms to the guidelines of the National Institutes of Health.

### Induction of obesity and CIA

Chicken type II collagen (CII) immunization was performed initially when mice weighed 25 g. Complete Freund’s adjuvant (CFA; Chondrex, Redmond, WA, USA) was prepared by grinding 4 mg of heat-killed *Mycobacterium tuberculosis* (H37Ra; Difco Laboratories, Detroit, MI, USA) mixing with 2 ml of incomplete Freund’s adjuvant (IFA; Chondrex). An emulsion was then formed by dissolving 4 mg ml^−1^ CII (Chondrex) overnight at 4 °C in 0.5 M acetic acid, followed by mixing the solution with an equal volume of CFA. The mice were injected intradermally in the tail. A booster injection was administered 14 days after the primary immunization. The score of arthritis severity in the joints of these mice was determined twice weekly; the arthritis score was recorded as the sum of the scores on a scale of 0–4.

### Metformin and Enbrel treatment

Metformin was obtained from Sigma-Aldrich (St Louis, MO, USA) and dissolved in saline. Mice were given 50 mg kg^−1^ of oral metformin daily for 13 weeks starting on day 7 after the first immunization. Enbrel (Pfizer, New York, NY, USA) was injected subcutaneously (SC) 3 times per week after the initial immunization. The Enbrel dose was 100 μg per mouse. Control mice were injected with saline.

### Histological assessment

Mouse joint, liver and interscapular BAT (iBAT) samples were obtained 13 weeks after immunization and fixed in 4% paraformaldehyde, decalcified in calci-clear rapid (National diagnostics), and embedded in paraffin. The joint, liver and iBAT were sectioned at a thickness of 6 μm, deparaffinized using xylene, dehydrated through a gradient of alcohol and then stained with hematoxylin and eosin (H&E), or Safranin O. The H&E-stained sections were scored for inflammation and bone erosion. Inflammation was scored according to the following criteria: 0=no inflammation, 1=slight thickening of the lining layer or some infiltrating cells in the underlying layer, 2=slight thickening of the lining layer plus some infiltrating cells in the underlying layer, 3=thickening of the lining layer, an influx of cells in the underlying layer, and the presence of cells in the synovial space, and 4=synovium highly infiltrated with many inflammatory cells. Cartilage damage was determined using Safranin O staining where the extent of cartilage damage was scored according to the following criteria: 0=no destruction, 1=minimal erosion limited to single spots, 2=slight-to-moderate erosion in a limited area, 3=more extensive erosion, and 4=general destruction. Immunohistochemistry (IHC) staining was performed using a Vectastain ABC kit (Vector Laboratories). The tissues were incubated with anti-IL-17 and anti-IL-6 antibodies (Santa Cruz Biotechnology Inc., SantaCruz, CA, USA) overnight at 4 °C. These primary antibodies were detected with a biotinylated secondary linking antibody for 40 min, followed by incubation with streptavidin–peroxidase complex for 1 h. The final color product was developed using 3,3′-diaminobenzidine (DAB) chromogen (DAKO, Carpinteria, CA, USA). Positive cells were counted, with the results expressed as the mean±s.d.

### Confocal microscopy

For immunostaining, 7 μm tissue sections of spleens were stained. To analyze the populations of T helper cells, we used Alexa 488 conjugated anti-CD4, PE-conjugated anti-IL-17, APC-conjugated anti-CD25, and PE-conjugated anti-Foxp3 antibodies (eBiosciences, San Diego, CA, USA). To analyze the populations of STAT, AMPK and mTOR, the samples were stained with Alexa 488 conjugated anti-CD4, PE-conjugated anti-phosphorylated STAT-3 tyrosine 705, PE-conjugated anti-phosphorylated STAT-3 tyrosine 727, anti-mTOR, anti-AMPK, and anti-FGF21 antibodies, and anti-rabbit IgG-PE secondary antibody. The nuclei were stained with 4′,6-diamidino-2-phenylindole. The stained sections were analyzed using a Zeiss microscope (LSM 510 Meta; Carl Zeiss, Oberkochen, Germany) at × 400 magnification. Positive cells were counted, and the numbers expressed as the mean±s.d.

### Biochemical analyses

Blood samples were collected from all treated and control mice at 13 weeks and stored at −70 °C until use. The levels of AST, ALT, HDL- and LDL-cholesterol were measured using commercial kits from Asan Pharmaceutical Co. (Hwangseong-gi, Gyeonggi-do, Korea).

### Analysis of gene expression by real-time quantitative PCR

Total RNA was extracted using TRIzol (Molecular Research Center, Cincinnati, OH, USA). Two micrograms of total RNA was reverse transcribed using the Superscript Reverse Transcription system (Takara, Shiga, Japan). Quantitative real-time PCR (qRT-PCR) was performed with LightCycler FastStart DNAmaster SYBR green I (Takara) fluorescent dye using an ABI PCR machine. Primers for FGF-21 (forward: GCATACCCCATCCCTGACTC, reverse: ACCACTGTTCCATCCTCC CT), IL-17 (forward: CCTCAAAGCTCAGCGTGTCC, reverse: GAGCTCACTTTTGCGCCAAG), IKBKE (forward: CCCAAAGTTCGTCCCTAAGGTTG, reverse: ATCAACGCCTGTCCATCCAGCA) and β-actin (forward: 5′-GAAATCGTGCGTGACATCAAAG-3′, reverse: 5′-TGTAGTTTCATGGATGCCACAG-3′) were designed using Primer Express (Applied Biosystems, Foster City, CA). The mRNA expression levels were normalized to those of β-actin.

### Murine T-cell isolation and differentiation

Spleen cell cultures were performed in RPMI 1640 medium supplemented with 5% FBS. To purify CD4^+^ T cells, the cells were incubated with CD4-coated magnetic beads and isolated using magnetic-activated cell sorting (MACS) separation columns (Miltenyi Biotec). Positively selected CD4^+^ T cells were stimulated with plate-bound anti-CD3 (0.5 μg ml^−1^), soluble anti-CD28 (1 μg ml^−1^; both from BD Biosciences), anti-interferon-γ (2 μg ml^−1^), anti-IL-4 (2 μg ml^−1^) antibodies, recombinant TGF-β (2 ng ml^−1^), and recombinant IL-6 (20 ng ml^−1^) (R&D Systems) for 3 days to achieve polarization of Th17 cells.

### Co-culture experiment of Th17 cells with BAT

Spleen Th17 cells were seeded in 24-well plates at 1 × 10^6^ per well in 5% RPMI, and BAT was layered onto Th17 cells. Culture plates were incubated at 37 °C for 72 h, and then, the culture supernatants were collected.

### BAT transplantation

BAT was removed from DBA1/J donor mice (age 7 weeks) and washed in sterile PBS. We then transplanted 0.2 g donor BAT into the subcutaneous dorsal region of CIA mice as quickly as possible. Recipient mouse spleen samples were obtained 5 weeks after the transplantation. The control group underwent the same procedure.

### Enzyme-linked immunosorbent assay

The amounts of IL-17 in culture supernatants derived from mice were measured by sandwich enzyme-linked immunosorbent assay (ELISA, R&D Systems). Alkaline phosphatase (Sigma-Aldrich) was used for color development. Absorbance was measured at 405 nm on an ELISA microplate reader (Molecular Devices).

### Measurement of IgG concentrations

Blood samples were taken from the orbital sinuses of mice, and the serum concentrations of IgG, IgG1 and IgG2a were measured using mouse IgG, IgG1 and IgG2a ELISA quantitation kits (Bethyl Laboratories), respectively.

### Intracellular staining and flow cytometry

Cells were stimulated with 25 ng ml^−1^ PMA (Sigma-Aldrich, St Louis, MO), 250 ng ml^−1^ ionomycin (Sigma-Aldrich) and Golgi Stop (BD Biosciences, San Diego, CA) in 5% CO_2_ at 37 °C for 4 h. Cells were stained with Percp-conjugated anti-CD4 antibody and APC-conjugated anti-CD25 Ab (BD Pharmingen) and then stained with APC-conjugated anti-IFN-γ, PE-conjugated anti-IL-4, FITC-conjugated anti-IL-17 or PE-conjugated anti-Foxp3 (all from eBiosciences), followed by fixation and permeabilization using the Cytofix/Cytoperm Plus Kit (BD Biosciences) according to the manufacturer’s instructions. All samples were processed with FACSCalibur (BD Pharmingen), and the data were analyzed using FlowJo software (Tree Star, Ashland, OR, USA).

### Statistical analysis

Statistical analyses were performed using GraphPad Prism 5 software. Differences between treatment groups were tested for statistical significance with the Mann–Whitney *U*-test. The results are expressed as the means±s.d. (or means±s.e.m.). The data were considered significantly different at *P*<0.05 (two-tailed).

## Results

### Metformin ameliorates the progression of CIA in obese mice

**Figure 1:**
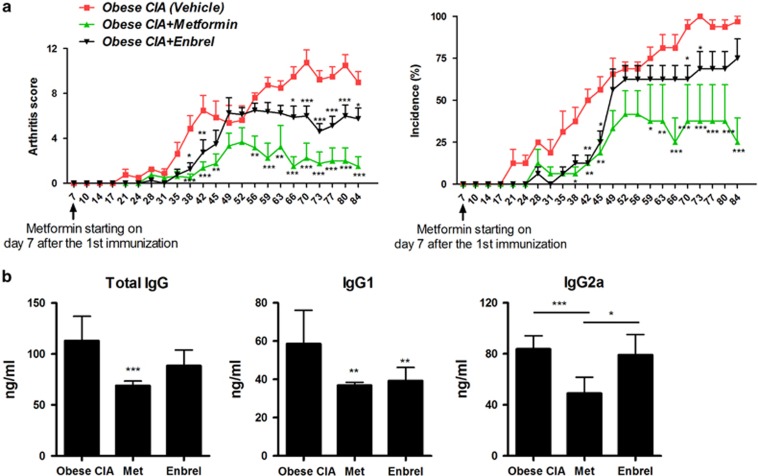
Treatment with metformin attenuated in obese collagen-induced arthritis (CIA) mice. (**a**) Reduction in the arthritis score and arthritis incidence in obese CIA mice treated with metformin. (mean±s.e.m. of five mice per group,*P<0.05, **P<0.01, ***P<0.001). (**b**) Levels of IgG, IgG1 and IgG2a were determined by enzyme-linked immunosorbent assay. (Mean±s.d. of five mice per group,*P<0.05.).

Compared to mice that received vehicle, metformin-treated mice had a significantly lower arthritis score during the entire experimental period. Treatment of mice with metformin also attenuated the progression of arthritis compared with mice receiving Enbrel (Figure 1a). The concentrations of total IgG, IgG1 and IgG2a in the serum of metformin-treated obese CIA mice were less than those in vehicle-treated and Enbrel-treated obese CIA mice (Figure 1b).

### The anti-inflammatory profile of metformin-treated mice

**Figure 2:**
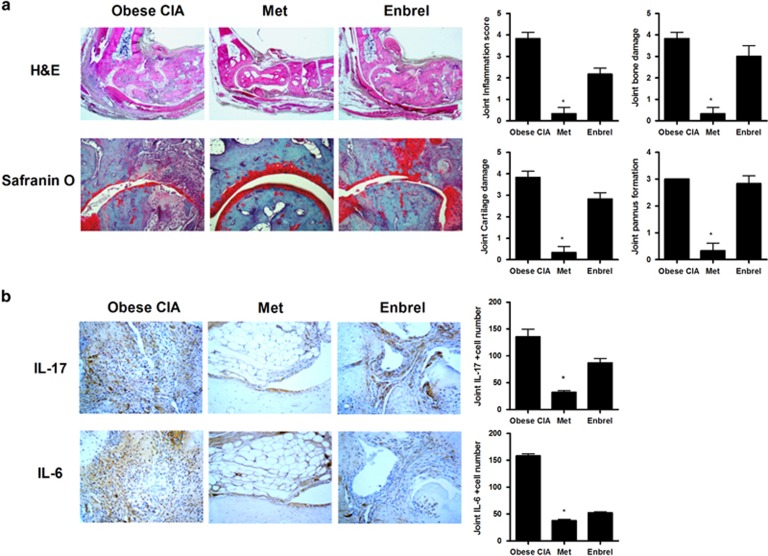
Effects of metformin on proinflammatory molecules in the joints of collagen-induced arthritis (CIA) and obese CIA mice. (**a**) Histologic features of the joints were stained with hematoxylin and eosin (H&E), and Safranin O. (**b**) Tissue sections from joints were immunohistochemistry stained with anti-IL-6 and anti-IL-17 antibodies. (Values are the mean ±s.d., *P<0.05, **P<0.01.)

Consistent with the arthritis score, minimal signs of inflammation were detected in the metformin-treated obese CIA mice, whereas extensive infiltration of immune cells was observed in vehicle- and Enbrel-treated obese CIA mice. Histological analyses also showed that joint destruction, bone and cartilage damage, and pannus formation were ameliorated in the metformin-treated obese CIA mice compared to vehicle- or Enbrel-treated obese CIA mice (Figure 2a). Additionally, the expression levels of IL-6 and -17 in the joints were significantly lower in the metformin-treated obese CIA mice than in the vehicle-treated obese CIA mice (Figure 2b).

### Counter-regulatory effects of metformin on Th17/Treg cells in obese CIA mice are associated with activation of STAT3

**Figure 3:**
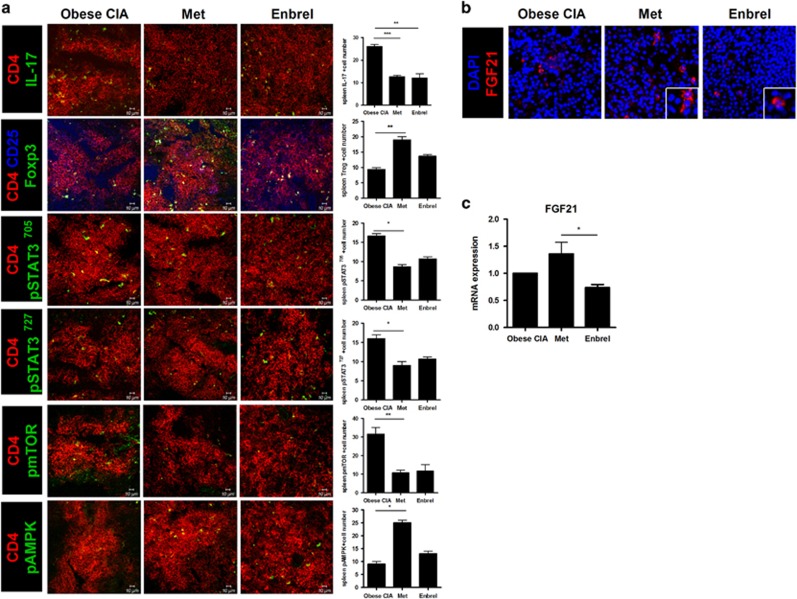
Metformin reduced STAT3 phosphorylation, decreased the frequency of Th17 cells within the population of CD4^+^ T cells and induced FGF21 expression in collagen-induced arthritis (CIA) and obese CIA mice. (**a**, **b**) Spleen tissues from each mouse were examined by immunofluorescence staining. Original magnification × 400. (**c**) Expression of FGF21 mRNA in isolated splenocytes was measured by real-time polymerase chain reaction, with the results normalized to the expression of β-actin mRNA. (Values are the mean±s.d., *P<0.05, **P<0.01.)

Confocal imaging showed significantly fewer CD4^+^IL-17^+^, CD4^+^pSTAT3 Tyr705^+^, CD4^+^pSTAT3 Ser727^+^ and CD4^+^pmTOR^+^ cells in the spleen tissue of the metformin-treated obese CIA mice than in that of the vehicle- or Enbrel-treated obese CIA mice. However, there were significantly more CD4^+^CD25^+^Foxp3^+^ and CD4^+^pAMPK^+^ cells in the spleen tissue of metformin-treated obese CIA mice than in that of vehicle- or Enbrel-treated obese CIA mice (Figure 3a). There was also a significant upregulation of FGF21^+^ cells in spleen tissue from metformin-treated obese CIA mice compared to the vehicle- or Enbrel-treated obese CIA mice (Figure 3b). The gene expression of FGF21 in the spleen was also significantly increased with metformin treatment (Figure 3c).

### Effect of metformin treatment on metabolic profiles in obese CIA mice

**Figure 4:**
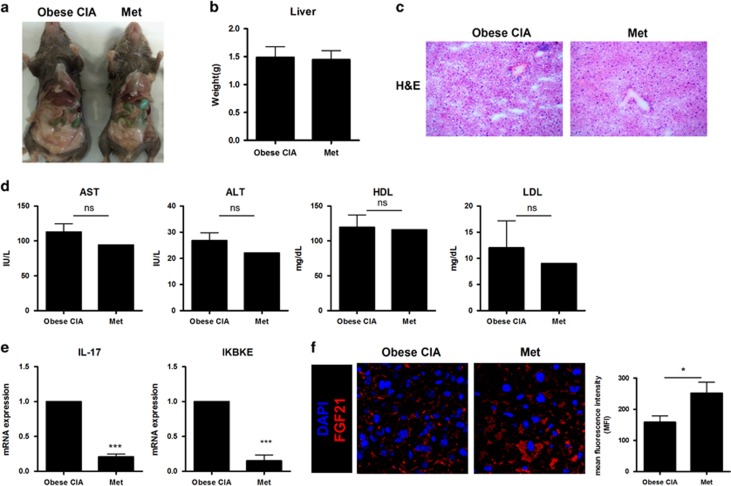
Metformin treatment ameliorated fatty liver and normalized metabolic profiles. (**a**, **d**) Obese collagen-induced arthritis (CIA) mice or metformin-treated obese CIA mice had a similar phenotype, but different metabolic profiles. (**b**) Liver tissues obtained from obese CIA and metformin-treated obese CIA mice were weighed. (**c**) Sections were stained with hematoxylin and eosin (h&e). Original magnification × 400. (**e**) Expression of IL-17 and IKBKE mRNA in isolated hepatocytes was measured by real-time polymerase chain reaction, with the results normalized to the expression of β-actin mRNA. (**f**) Liver tissues from obese CIA and metformin-treated obese CIA mice were examined by immunofluorescence staining. Original magnification × 400. (Values are the mean±s.d., *P<0.05, **P<0.01, ***P<0.001.)

Metformin treatment caused no changes in weight (data not shown) or other macroscopic changes (Figure 4a). The weight of the liver also did not change with metformin treatment (Figure 4b). However, H&E staining showed a decrease in the immune cell infiltration of the liver among metformin-treated obese CIA mice compared to vehicle-treated obese CIA mice (Figure 4c). Metformin slightly reduced the levels of AST, ALT and LDL in the serum from obese CIA mice. The HDL level did not change with metformin treatment (Figure 4d). The levels of IL-17 and IKBKE mRNA in the liver of metformin-treated obese CIA mice were significantly downregulated compared to those in the vehicle-treated obese CIA mice (Figure 4e). However, treatment with metformin significantly increased the expression of FGF21 in the liver of obese CIA mice (Figure 4f).

### Effect of metformin treatment on BAT regulation in obese CIA mice

**Figure 5:**
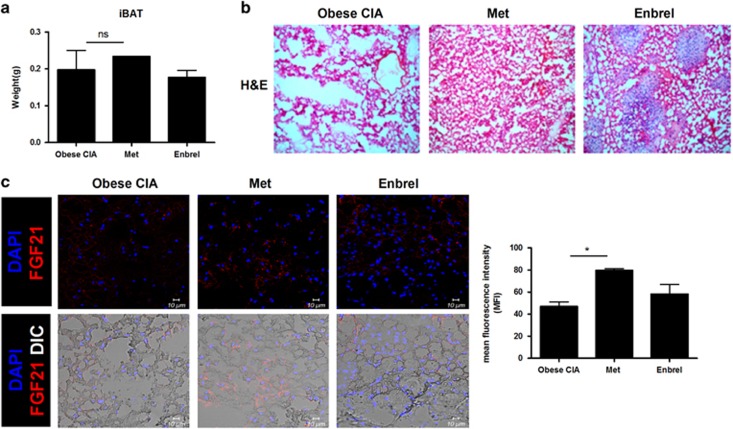
Brown adipose tissue was upregulated by metformin treatment. (**a**) Weight of brown fat obtained from obese CIA mice or metformin- or Enbrel-treated obese CIA mice. (**b**) Histologic features of the brown fat tissues stained with hematoxylin and eosin (h&e). Original magnification × 200. (**c**) Brown fat tissues from each mouse were stained for FGF21^+^ cells. Original magnification × 400.

There was an increase in the weight of BAT in obese CIA mice treated with metformin, but it was not significant (Figure 5a). Metformin treatment also reduced the infiltration of immune cells in the BAT of obese CIA mice (Figure 5b). FGF21 production in the BAT of obese CIA mice was also increased with metformin treatment (Figure 5c).

### Effect of co-culture and transplantation with BAT on the suppression of Th17 and IL-17

**Figure 6:**
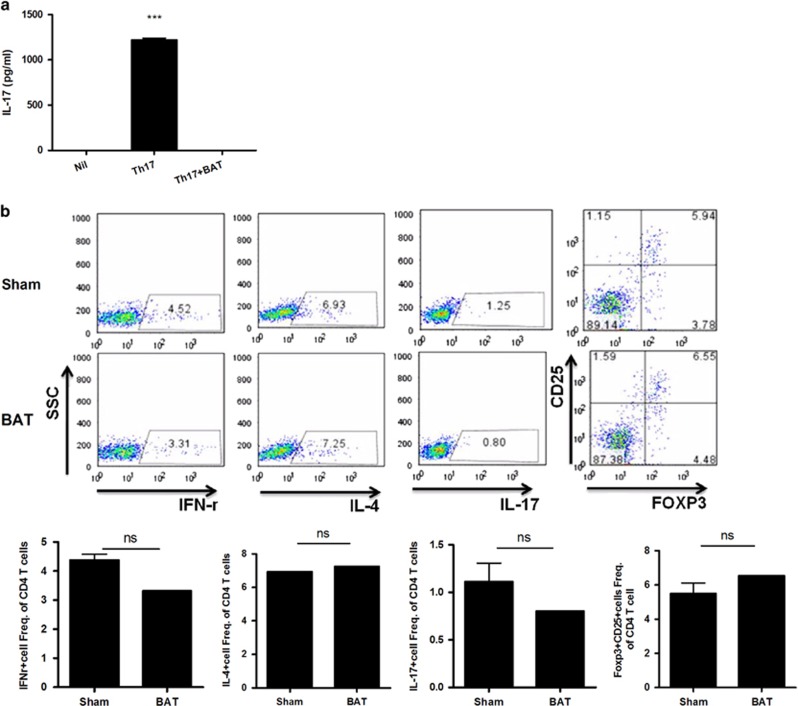
BAT reduced the activation of T cells and inhibited Th17 differentiation. (**a**) Splenocytes were cultured in Th17 conditions and co-cultured with BAT. IL-17 levels in the culture supernatants were measured by ELISA. (**b**) The differentiation of helper T cells and regulatory T cells obtained from the spleens of mice that received BAT transplants.

IL-17 levels significantly decreased in the culture medium obtained from Th17 cells co-cultured with BAT (Figure 6a). Th1 and Th17 cell differentiation was also decreased in the spleen of mice transplanted with BAT, but the difference was not significant (Figure 6b).

## Discussion

Metformin is known to be an effective treatment for type 2 diabetes and obesity. Recently, metformin was revealed to have an anti-arthritic effect on experimental autoimmune arthritis.^21^ To the best of our knowledge, the mechanism of metformin’s action and its activity on BAT in obese CIA mice has yet to be documented. In this investigation, the therapeutic functions of metformin in an obese CIA mouse model were observed, and the findings suggested the possibility of using metformin in therapeutic strategies against RA associated with obesity.

An important observation was the therapeutic efficacy of metformin in obese CIA mice and its association with the reciprocal Th17/Treg balance and the upregulation of BAT differentiation. The downregulation of Th17 and the upregulation of Tregs is an important therapeutic process in experimental autoimmune arthritis.^21, 24, 25^ A high-fat diet has been shown to induce the downregulation of BAT while increasing markers of fatty liver, including AST and ALT.^26^ By contrast, the upregulation of BAT has been shown to reduce the levels of plasma ALT and AST.^27^ It is also widely believed that BAT has therapeutic potential in the treatment of metabolic disorders.^13^ Here it was found that metformin reduces obesity and has an anti-arthritic effect via inducing BAT and restoring the reciprocal balance between Th17 and Tregs. In addition, metformin reduced the infiltration of immune cells in the liver and BAT. We observed that transplantation of BAT decreased Th17 cell differentiation, while a co-culture with BAT reduced IL-17 expression *in vitro*. These results suggest that metformin can decrease the inflammatory response in RA associated with metabolic disorders.

Metformin decreased the expression of pSTAT3 and mTOR and enhanced pAMPK expression. The inflammatory response and the expression of proinflammatory cytokines, including IL-6 and -17, are induced by pSTAT3.^28^ By contrast, AMPK inhibits the activation of mTOR, which in turn leads to enhanced expression of pSTAT3.^29, 30^ It has been demonstrated that the IL-6 level in the synovial fluid and blood of RA patients is excessively high and is positively correlated with joint destruction.^31, 32^ IL-17 is also produced at high levels in the synovium and synovial fluid of RA patients.^33^ In this study, a significant downregulation of IL-6 and -17 was found in the joint tissue of metformin-treated obese CIA mice. Additionally, the expression levels of mTOR and pSTAT3 were reduced with metformin treatment. Since the inhibition of proinflammatory cytokines and pSTAT3 expression plays a key role in the suppression of inflammation, our results reveal that metformin has anti-inflammatory activity in obese CIA mice.

Previously, metformin was found to downregulate the levels of obesity-related factors, including cholesterol and LDL, but to promote FGF21 expression in obese mice.^23^ Since FGF21 has been shown to be a key regulator of obesity,^14^ enhancing FGF21 expression has great potential therapeutic efficacy. FGF21 also attenuates obesity-mediated inflammation. Recently, treatment with FGF21 slowed the progression of obesity by downregulating ALT, AST and IL-6.^34^ There are recent reports that FGF21 attenuated CIA development, decreased inflammatory cytokine production and diminished the Th17-IL-17 axis via the STAT3 pathway.^18, 35^ We observed that metformin reduced the levels of inflammatory mediators, such as IKBKE, which is a therapeutic target for obesity-associated inflammation^36^ and a means to increase FGF21 production in the liver and BAT. Therefore, our observations suggest that metformin has an anti-obesity effect, as well as an inflammatory effect, in obese CIA mice.

Previous research has documented the ability of metformin to induce FGF21 production,^37^ as well as attenuate the progression of obesity, thereby ameliorating dysregulated metabolic function.^23, 38, 39^ On the other hand, metformin decreases FGF21 expression. It has been suggested that treatment with metformin downregulates the FGF21 level in the plasma of type 2 diabetes mellitus patients.^40, 41^ However, the effect of metformin on FGF21 levels is associated with an anti-inflammatory effect. Metformin has also been shown to reduce the expression of high-sensitivity C-reactive protein, which is an inflammatory indicator that can demonstrate the progression of metabolic disorder in the plasma of type 2 diabetes mellitus patients.^41^ Metformin might decrease FGF21 levels by alleviating inflammation in type 2 diabetes mellitus patients.

Our findings demonstrate the possibility of metformin treatment for autoimmune arthritis associated with metabolic disease. Our study suggests that metformin ameliorates the inflammatory response and decreases obesity progression and may be a therapeutic candidate for RA associated with metabolic disorders.
